# How Patients Who Are Transported by Ambulance Experience Dyspnea and the Use of a Dyspnea Scale: A Qualitative Study

**DOI:** 10.3390/healthcare10071208

**Published:** 2022-06-28

**Authors:** Stine Ibsen, Birgitte Schantz Laursen, Erika Frischknecht Christensen, Ulla Møller Weinreich, Søren Mikkelsen, Tim Alex Lindskou

**Affiliations:** 1 Centre for Prehospital and Emergency Research, Aalborg University and Aalborg University Hospital, 9000 Aalborg, Denmark; efc@rn.dk (E.F.C.); tim.l@rn.dk (T.A.L.); 2 Department of Physiotherapy, University College of Northern Denmark, 9000 Aalborg, Denmark; 3 Clinical Nursing Research Unit, Aalborg University Hospital, 9000 Aalborg, Denmark; bisl@rn.dk; 4 Sexology Research Centre, Department of Clinical Medicine, Aalborg University, 9000 Aalborg, Denmark; 5 Clinic of Internal and Emergency Medicine, Department of Emergency and Trauma Care, 9000 Aalborg, Denmark; 6 Department of Respiratory Diseases, Aalborg University Hospital, 9000 Aalborg, Denmark; ulw@rn.dk; 7 The Clinical Institute, Aalborg University, 9000 Aalborg, Denmark; 8 The Prehospital Research Unit, Region of Southern Denmark, 5000 Odense, Denmark; soeren.mikkelsen@rsyd.dk

**Keywords:** dyspnea, prehospital emergency care, patient’s experience, breathing difficulties, interview

## Abstract

Approximately 7% of all dispatched ambulances in Denmark are for patients for whom breathing difficulties are the main cause for using ambulance services. Objective measurements are routinely carried out in the ambulances, but little is known of the patients’ subjective experience of dyspnea. The purpose of this study was to investigate how patients with acute dyspnea, transported to hospital by ambulance, experience their situation, along with their experience of the use of a dyspnea scale. The study was carried out in the North Denmark Region. Transcribed patient interviews and field notes were analyzed and interpreted with inspiration from Paul Ricoeur. For interviews, we included 12 patients with dyspnea who were transported to the hospital by ambulance: six women and six men all aged 60 years or above. Observations were made over six ambulance transports related to dyspnea. Three themes emerged: “anxiety”, “reassurance in the ambulance” and “acceptance of the dyspnea measurements in the ambulance”. Several patients expressed anxiety due to their dyspnea, which was substantiated by observations in the ambulance. The patients expressed different perspectives on what improved the situation (treatment, reassurance by ambulance professionals). The patients and the ambulance personnel were, in general, in favor of the dyspnea scale.

## 1. Introduction

Dyspnea is defined as a subjectively experienced symptom of breathing discomfort and is highly distressing [1]. It is multidimensional by nature, as both physical, psychological, and social aspects affect, and are affected by, dyspnea [2,3]. Due to the multidimensional nature of dyspnea, the assessment is complicated, and communication is difficult because of patients’ shortness of breath, especially in an acute, stressful situation [4]. Dyspnea is a symptom of several underlying conditions. It is a frequent symptom in the prehospital setting and is associated with high mortality [1].

Approximately 7% of all dispatched ambulances in Denmark are for patients for whom breathing difficulties are the main cause for calling an ambulance [5]. Objective measurements, such as blood oxygen saturation and respiratory frequency, are routinely carried out in the ambulances, but little is known of the patients’ subjective experience of dyspnea. A previous Danish study addressed how patients transported to the hospital by ambulance experience the intensity of dyspnea in the ambulance and whether they experience relief of symptoms before hospital arrival. This was done by implementing a 0 to 10 verbal rating scale for acute dyspnea to use in the ambulance. The study demonstrated that patients transported to the hospital by ambulance reported an initial high intensity of dyspnea (median of 8 (interquartile range 6–10)), which decreased after prehospital treatment [6]. Likewise, previous prehospital studies rely primarily on quantitative data [7,8]. However, dyspnea needs to be understood from the patients’ perspective to optimize prehospital care for these patients. A recent qualitative study from Sweden describes how dyspnea was experienced by patients prior to prehospital care. The study revealed that it is only when patients experience extreme existential fear and severe anxiety that they seek emergency help [9]. This emphasizes that the healthcare personnel need to be both efficient in treating and emotionally responsive, intending to alleviate severe anxiety. Therefore, the qualifications of the paramedics who are the first to attend and treat the patient should include not only medical knowledge but also knowledge of psychological care for a patient who experiences severe dyspnea in order to implement this in a prehospital setting. The patient experience of ambulance transport to hospital due to dyspnea is not well described in previous research.

Thus, with this study, we aimed to investigate how patients with acute dyspnea, transported to the hospital by ambulance, experience their situation when calling the emergency number and during the ambulance run. Furthermore, we aimed to investigate the patients’ thoughts and experience with the dyspnea scale.

## 2. Materials and Methods

To fulfill our aim, we carried out a focused ethnography-inspired study in the North Denmark Region. Observational data were gathered from inside ambulances during runs from three different ambulance station locations. In addition to this, patient interviews were performed. Focused ethnography was chosen as the methodology, as this method is applicable when the research concerns both problem-focused and context-specific frameworks [10]. The method was also chosen due to the communication limitations of patients experiencing dyspnea, as it combines observations of patients in an acute situation with interviews when a patient is in a more stable phase of the acute situation. The intention was to explore the patients’ experiences of their situation when transported to a hospital due to acute dyspnea. The reporting of the study is carried out in accordance with the Consolidated Criteria for Reporting Qualitative Research (COREQ) [11].

The study was conducted in the North Denmark Region, which is home to approximately 587,000 citizens [12]. Danish healthcare, including prehospital, is tax funded and therefore free of charge for patients. Emergency calls of a medical nature are handled by the police and forwarded to healthcare professionals. Ambulances, manned by two ambulance professionals, rapid response vehicles with paramedics or prehospital anesthesiologists, and helicopter emergency medical services, are available to be dispatched. Patients who receive an ambulance after an emergency call receive treatment and are transported to a hospital or released on the scene [13]. The North Denmark Region has two hospitals with emergency departments, the North Denmark Regional Hospital in Hjørring and Aalborg University Hospital in Aalborg.

This study is a supplement to a previous Danish project concerning patients with dyspnea transported to the hospital by ambulance [6,14,15]. In that study, a verbal numerical rating scale (0–10) was implemented. The scale is designed to assess dyspnea and with the scale, the patient is asked, “*On a scale from 0 to 10, where 0 is no breathing difficulties and 10 is the worst possible breathing difficulties imaginable, how are you experiencing your breathing now?*” [6].

The interviews and observations were conducted separately over two months between May and June 2018, during daytime work hours, and they included two different participant groups. The observed patients were therefore not the same patients who were interviewed. For the interviews, we included a convenience sample of patients with dyspnea transported to the hospital by ambulance. Patients were included for interviews in the two emergency departments in the region. No repeated interviews were carried out. We excluded patients aged <18 years and patients not able to speak and understand Danish. The interviews and the observations were carried out by the last author, who is a registered nurse, Master of Science, and was a PhD fellow at the time. He did not receive any training before data collection but was guided by one of the supervisors who is an experienced qualitative researcher [16]. He approached eligible patients and provided verbal and written information on the purpose of the study and himself. The interviewing author did not present himself as a healthcare professional to the patients to limit possible white coat bias. The interest in the research topic was related to the author’s PhD topic at the time, which concerned patients with dyspnea. The study was carried out on pre-selected days where the interviewer participated in the ambulance runs. The study population, therefore, consisted of the patients who experienced dyspnea which made an ambulance run necessary on the given days. The patients were approached face to face by the author as soon as their medical situation was stable, i.e., no urgent treatment required, as assessed by healthcare personnel at the emergency department.

Data were collected through semi-structured interviews based on an interview guide to ensure consistency in the interviews [17].

The interview guide is available in Table 1.

The semi-structured interviews allowed participants to express thoughts that they considered important for their experience. The interviews were followed up with additional questions if required to fully understand the patient’s perspective. Although the interviewing author had some previous experience with interviews, formally, he should be considered a novice. All interviews were conducted in the clinical setting of the departments and paused for any medical needs or if any healthcare professionals were present in the room. Data saturation was continuously assessed in the data collection period.

The interviewing author also carried out observations as he accompanied ambulance professionals at the ambulance stations and during the ambulance runs. For the observations, we included a convenience sample, i.e., ambulance runs related to breathing difficulty. The observations were carried out in the areas related to the catchment areas of two specific emergency departments in the Region of Northern Denmark. When not on an ambulance run, the author conducted informal interviews with the ambulance professionals and establishes a frame of reference concerning patients with dyspnea. During ambulance runs, the author was present in the background at the scene. When in the ambulance with the patient, he was located behind the stretcher facing the ambulance professionals and the patient. The time following an ambulance run facilitated informal interviews regarding patients suffering from dyspnea. Field notes were used for observations as well as informal interviews with the ambulance professionals.

All interviews were audio recorded and later transcribed verbatim by the interviewing author. The included patients did not receive the transcribed interviews for comments. The transcribed patient interviews and field notes were analyzed and interpreted by the interviewing author and the first author with inspiration from Paul Ricoeur [18,19]. First, data were read and re-read naively, to gather an understanding. secondly, data were analyzed and structured according to deduced themes. The themes were identified within the explicit meanings of the data, and we did not look for anything beyond the patients’ statements and what was observed. These two initial steps were done individually by the two authors, and the deduced themes were collated to ensure consistency. In case of disagreement between authors, the deduced themes were discussed, and a common understanding was reached. Finally, the deduced themes were critically interpreted in thematic analysis by both authors in an attempt to theorize the broader meaning and the implications [19].

The interviewed patients were not presented with the deduced themes for comments. The transcribed data were anonymized before analysis, which was carried out with the use of Microsoft Word 2016.

The study was approved by the Danish Data Protection Agency (North Denmark region record number 2008-58-0028 and project ID number 2017-128).

Participants in the interviews received verbal and written information regarding the study before the interview and were asked to sign an informed consent. They were informed that they had the right to withdraw from the study at any point without consequences. This is in accordance with Danish legislation where interviews and observations do not require approval from the Committee on Health Research Ethics or other instances [20]. The involved parties of this study report no conflicting interests.

The interviews and the observations included two different participant samples, and as such, no patient was both observed and interviewed. We included 12 patients with dyspnea transported to the hospital by ambulance, 6 women and 6 men all aged 60 years or above in the interviews. Observations were made over 6 ambulance runs related to breathing difficulties. There were no dropouts or rejections to participate in the study. For two of the interviews, a family member was present. The interviews lasted on average of 7 min. In general, the patients gave concise answers, and one patient expressed a need to concentrate on breathing and therefore nodded to answer questions. Data saturation was obtained from the 12 interviews, as no new themes were deduced.

## 3. Results

Three themes emerged: “anxiety”, “reassurance in the ambulance” and “acceptance with dyspnea measurements in the ambulance”.

In Section 3.1, the findings for each theme are described and illustrated with excerpts from the interviews. All interviews were carried out in Danish. The excerpts provided in the text are translated, aimed to carry the same meaning in English, e.g., by avoiding local idioms, and therefore are not verbatim translation. Patients are identified with a character ID (A to L)

### 3.1. Anxiety

Through informal interviews with ambulance professionals during the observations, the ambulance personnel appeared aware of the situation of the patients in question. They described fear and anxiety as interpretation of the patients’ experiences. Observation of the patients during the ambulance run showed patients gasping for air, hands reaching out and grabbing ambulance personnel, and searching eyes. There was no doubt about the patients’ anxiety in the acute situation. However, some patients had a more calm appearance, heaving for air but responding to questions in a calm manner, despite difficulties speaking. Through the interviews, several of the patients expressed that they felt anxiety and were nervous. Thoughts about dying were also present in one patient.

*“You see, people who can’t breathe panic. They always do”*

(A)

*“I was a little nervous, I really was”*

(C)

*“Then you think, well, you are probably going to die. Well, that thought crosses your mind; I may as well be honest”*

(D)

The anxiety and the thoughts of death could indicate that the patients postpone seeking medical help until the situation is unmanageable and prompts an emergency call.

The patients expressed different coping strategies concerning their anxiety. One patient expressed that previously learned breathing technics helped the patient relax, while others mentioned collaboration with the ambulance personnel.

*“Then I said to one of the ambulance personnel, now it is as if it is worse. Then he gave me a little more (oxygen), and I was transported here (emergency department)”*

(D)

*“Over time you learn some breathing exercises. So, breathing using your diaphragm instead of your lungs relaxes you more.”*

(A)

### 3.2. Reassurance in the Ambulance

Despite the level of anxiety present among the patients, a reassurance or improvement of the situation occurred in the ambulance. The ambulance professionals presented themselves to the patients on the scene with a professional calm nature, and they continuously communicated and calmed the patient during treatment. For the majority of the observations, the patients’ vital signs did not improve during the ambulance run, e.g., respiratory rate and blood oxygen saturation remained the same (observed on monitoring equipment and through informal interviews with the ambulance professionals). However, the patients appeared more calm and secure in their verbal responses, and the anxious appearance lessened. This was also expressed in their subjective experiences:

*“It did not take long before very slowly… -You calmed down? Yes. But it takes a long time before you are completely calm”*

(H)

*“It did not take long for my breathing to become normal again (After entering the ambulance)”*

(A)

The patients expressed different perspectives on what improved the situation. Some patients mentioned specifically oxygen therapy

*“It was fine because I got oxygen and it helped immediately”*

(F)

*“It was much better travelling with the ambulance because then I got oxygen, and the breathing (frequency) went down“*

(A)

*“I felt it worked when I got oxygen”*

(G)

Another commonly mentioned improvement was the ambulance professionals themselves.

*“They (ambulance personnel) explained what they did and all. I thought it was nice”*

(J)

Getting into the ambulance made most of the patients feel better even though their vital signs remained unchanged. This may indicate that the ambulance personnel have skills and ability to calm the patients psychologically. This is indicated by the patients’ feeling of reassurance, as the ambulance personnel took responsibility for their condition and explained the situation. Overall, this could be interpreted as the patients feeling that the situation became more comprehensible as the ambulance personnel made their condition understandable and predictable.

One patient expressed impatience and frustration, as the ambulance professional did not immediately drive to the hospital but monitored and treated the patient before departure.

*“Well, I was annoyed that we could not just get going, that is, they had to fix things… I just had to get in to get treatment.”*

(E)

In this situation, the patient experienced the care and treatment provided by the ambulance professionals as a delay.

### 3.3. Acceptance with Dyspnea Measurements in the Ambulance

Both the patients and the ambulance personnel were asked about their experience with the dyspnea scale. The ambulance personnel were in favor of the dyspnea scale, e.g., in an situation where a patient rated a high value on the dyspnea scale both before and following drug administration, allowing the ambulance personnel to act on the assessment.

The patients who had used the dyspnea scale were, in general, also in favor of the scale and found it easy to rate their dyspnea.

*“-Were you able to answer? Yes, I was at a number four. -So it was not so bad? No. Number ten is the highest. (when further asked if it was difficult to assess) It was no problem”*

(B)

*“(Asked if ambulance personnel asked to assess dyspnea) Yes they did, and it was a number 9. -Did you find it hard to assess? No, it could not be much worse”*

(K)

On the other hand, some patients found it difficult to convert their dyspnea into a specific number.

*“It is not (easy to assess). It is the same as if your leg hurts, how much does it hurt? It is 10 when it falls off… But I have to say when I said 9 (on dyspnea scale) I felt really ill”*

(F)

A single patient directly said it was hard to use the scale.

*“It was, like, I just said something. It was actually a little hard to answer.”*

(E)

Patients who were not asked to assess their dyspnea in the ambulance were asked whether they were familiar with the type of scale/question. All patients knew the similar questions asked regarding pain. They reported similar responses regarding difficulties in choosing a specific number when not at either of the extreme ends.

## 4. Discussion

This study aimed to explore how patients who are transported by ambulance experience their situation when calling the emergency number and are transported to the hospital. Furthermore, we aimed to investigate the patients’ thoughts and experience with the dyspnea scale. Three themes were identified. These were “*anxiety*”, “*reassurance in the ambulance*” and “*acceptance of dyspnea measurements in the ambulance*”. Several of the patients expressed fear and anxiety due to their dyspnea, which was substantiated by observations in the ambulance. Observations in the ambulance revealed reassurance of the patients. These observations were supported by the patients’ statements. The patients expressed different perspectives on what improved the situation, including oxygen therapy and being reassured by ambulance professionals. One patient expressed that previously learned exercise helped the patient relax. Both the patients and the ambulance personnel were, in general, in favor of the dyspnea scale. A single patient expressed that it was hard to use.

The qualitative study design made it possible to explore the patients’ experiences of dyspnea and their experience of the situation when transported to the hospital by ambulance. The interviews were conducted in the Emergency department immediately after hospital admission. This meant that the included patients were affected by their dyspnea and the situation, which resulted in limited words and short interviews, on average of only 7 min. However, this also meant that the patients’ experiences were clear in memory.

The included patients for the interviews were all 60 years of age or above. Most patients transported with ambulance due to acute dyspnea are older, which is also characteristic of the study population in this study. Even so, it would be relevant for future studies to include the perspectives from younger adults, as their experiences may be different from the older population. Additionally, we did not collect data on what caused the dyspnea or whether the patients had experienced dyspnea at an earlier time. Patients with different underlying diseases or earlier experiences may have different experiences in the acute situation. Socioeconomic status may likewise have an impact. These aspects would be relevant to explore in future studies.

The included patients all suffered from moderate to severe dyspnea, which led to hospitalization following the ambulance run. As such, the study did not include patients with very severe dyspnea who were unable to communicate during the ambulance run. One of the aims of this study was, however, to use the momentum to talk to the patients whilst they were in the experience, which has not previously been described in the literature. It was considered to include patients with very severe dyspnea and interview them later in the cause of the acute event; this would however have compromised our focus, and it is, furthermore, well established that patients have cognitive disability following the event of very severe dyspnea [21]. The subjective experience of dyspnea may be affected by other factors, such as socioeconomic status and previous diseases. These data were unfortunately not obtained from the patients.

As the patients had to be present in the Emergency departments and stabilized, it is reasonable to assume the most acute critically ill patients were not included, as they would have been transferred to intensive care units. Likewise, the least acute patients may also have eluded inclusion, as they were released from the department before an interview could be carried out, e.g., young patients admitted due to smoke inhalation. For the observational studies, the interviewing author accompanied the ambulance professionals on the ambulance runs. This meant that patients were included at random as a convenience sample and not strategically selected. Some selection bias could be assumed regarding the above. However, we were able to carry out interviews and observations, despite the limitations of the acute setting.

Our study showed that patients who call an ambulance due to dyspnea experience severe anxiety. These results correspond to what has previously been shown in a Swedish qualitative study, where patients with dyspnea described severe anxiety and a struggle for survival before prehospital care [9].

Togher et al. found that patients who use emergency medical services such as an ambulance are anxious regarding their health, wish for reassurance from the ambulance personnel, and are further reassured by professional behavior [22]. This corresponds to our findings, as most of the patients expressed reassurance in the ambulance due to the ambulance personnel. A Swedish study found that helicopter emergency medical services trauma patients expressed the feeling of “being taken seriously” and as a “worst-case”, it enabled them to trust the helicopter personnel [23]. Although the patients in our study were transported in an ambulance, the observed reassurance in the ambulance could be caused by the patients feeling the ambulance personnel took them seriously.

A Danish study, which conducted interviews 2 weeks after the patients had contacted the Emergency department due to dyspnea, found that patients, regardless of familiarity with dyspnea, worked out self-management strategies and stayed home for as long as possible, seemingly postponing seeking medical care [24]. Another qualitative study exploring the experience of patients with advanced COPD after a life-threatening event found that participants expressed confidence in the hospital to reduce their physical symptoms and related anxiety. Acute hospital care was often seen as a place of security [25]. Likewise, this was also observed in our study as the patients’ anxiety noticeably decreased during the period from ambulance professionals’ arrival at the patient to the ambulance arrival at a hospital. This occurred both for patients who had an improvement in vital signs as well as patients who had no improvement in their acute medical situation.

The patients in our study revealed different levels of anxiety along with different perspectives on what caused the reassurance in the ambulance. This indicates that the patients have different protective psychological mechanisms, including the extent of sense of coherence (SOC). Antonovsky conceptualizes SOC as a person’s ability to use existing and potential resources to combat stress and promote health, and it is measured based on one’s perception of manageability, meaning and comprehensibility. In general populations, stronger SOC was found to be associated with decreased mortality, even after controlling for sociodemographic and established risk factors [26,27,28]. This indicates that people suffering from dyspnea may benefit from interventions focusing on different coping strategies and on how to strengthen their SOC. Potentially, ambulance personnel facilitating the patients’ coping strategies may increase the patient psychological status during the ambulance run. A study from Denmark showed that patients with breathing difficulties were likely to use ambulances repeatedly [29]. It would be relevant to gain insight into the patients’ use of coping strategies before the emergency call as well to understand what leads to the emergency call. A study investigating the perceptions of the adequacy of dyspnea self-management strategies in persons with COPD found that most subjects used activity modification, decreasing activities, mowing slower and keeping still as a strategy to control dyspnea [30].

We found that several patients felt reassured in the ambulance. This corresponds to our previous study, which found that 76% of patients had a dyspnea scale score median of 8, which decreased to 4 during the ambulance run [6]. However, the patients’ improvement could be due to several factors, most of all the medical treatment. Yet a patient’s unconcerned approach does not necessarily reflect the patient’s situation, as it is an acute medical severe situation associated with a high risk of mortality [31]. The majority of observed patients also displayed greater levels of anxiety than expressed by the interviewed patients. It might be possible that the patients downplayed their anxiety in the interviews, for instance, due to the time passed since the incident, the improved medical situation, or to distance themselves from the unpleasantness.

The patients and ambulance personnel in our study were, in general, in favor of the dyspnea scale. In our previous study, we found the patients most frequently had a change of two to four points on the dyspnea scale, which were measured at ambulance arrival at the patient’s home and at hospital arrival. The flooring and ceiling effects were less than 9% [6]. Combined with the current findings, this suggests that the dyspnea scale, in general, is easy to use for the patients, although whether to rate 6 or 7 might be difficult. The difficulties of distinguishing between two successive values have likewise been reported for verbal numeric scales for pain [32,33].

## 5. Conclusions

The patients described and showed an experience of anxiety when experiencing acute dyspnea. However, a reassurance or improvement of the situation occurred in the ambulance. The patients expressed different perspectives on what caused the reassurance, but treatment and the ambulance personnel’s ability to calm the patient were emphasized. Different coping strategies were used by the patients to cope with their dyspnea. Further investigations are needed to evaluate whether patients suffering from dyspnea due to specific diseases have different perspective on their situation.

The ambulance professionals and most of the patients in this study were in favor of the dyspnea scale and found it easy to rate their dyspnea.

## Figures and Tables

**Table 1:** Interview guide.

Introduction and Purpose	Presentation of the Interviewer, the Purpose and Presentation of the Study. Information Regarding Recording the Interview, Anonymization and the Option to Withdraw Consent
Themes	Research Questions
**Introduction to the situation**	*What happened in the time before you experienced your breathing difficulties?*
	*Have you experienced it before?*
	*Was anyone with you?*
**Experience in the ambulance**	*How did you experience the situation in the ambulance?*
	*Did you feel scared, confused, nervous, or calm?*
	*Did anything calm and reassure you?*
	*How did you experience the ambulance personal?*
**Dyspnea scale**	*Were you asked to score your breathing difficulties on a scale from 0 to 10 in the ambulance?*
	*Do you know of a similar scale? (if not asked to score using the dyspnea scale)*
	*How do you understand the scale?*
	*How do you experience the use of the scales?*
**Debriefing**	*Ending the interview, ensuring there is nothing else the patient would like to add.*

## Data Availability

Not applicable.
